# Increased Prevalence of Autoimmune Gastritis in Patients with a Gastric Precancerous Lesion

**DOI:** 10.3390/jcm12196152

**Published:** 2023-09-23

**Authors:** Xiaopei Guo, Marco W. J. Schreurs, Fleur E. Marijnissen, Michiel C. Mommersteeg, Stella A. V. Nieuwenburg, Michail Doukas, Nicole S. Erler, Lisette G. Capelle, Marco J. Bruno, Maikel P. Peppelenbosch, Manon C. W. Spaander, Gwenny M. Fuhler

**Affiliations:** 1 Department of Gastroenterology and Hepatology, Erasmus MC, 3015 GD Rotterdam, The Netherlands; x.guo@erasmusmc.nl (X.G.); f.marijnissen@erasmusmc.nl (F.E.M.); m.mommersteeg@erasmusmc.nl (M.C.M.); s.nieuwenburg@erasmusmc.nl (S.A.V.N.); m.bruno@erasmusmc.nl (M.J.B.); m.peppelenbosch@erasmusmc.nl (M.P.P.); v.spaander@erasmusmc.nl (M.C.W.S.); 2 Department of Immunology, Erasmus MC, 3015 GD Rotterdam, The Netherlands; 3 Department of Pathology, Erasmus MC, 3015 GD Rotterdam, The Netherlands; m.doukas@erasmusmc.nl; 4 Department of Biostatistics, Erasmus MC, 3015 GD Rotterdam, The Netherlands; n.erler@erasmusmc.nl; 5 Department of Epidemiology, Erasmus MC, 3015 GD Rotterdam, The Netherlands; 6 Department of Gastroenterology and Hepatology, Meander Medical Center, 3813 TZ Amersfoort, The Netherlands; lg.capelle@meandermc.nl

**Keywords:** autoimmune gastritis, gastric precancerous lesions, gastrin 17, pepsinogen

## Abstract

**Background**: Autoimmune gastritis (AIG), characterized with the presence of anti-parietal-cell antibodies (APCA), is a risk factor for gastric cancer. However, AIG may go underdiagnosed, especially in the case of *H. pylori* infection and the presence of gastric precancerous lesions (GPL), due to the ambiguous pathology and delayed symptom onset. **Aim**: Investigate the prevalence and characteristics of AIG in GPL patients. **Methods:** Prevalence of AIG was determined with the presence of APCA in patients with GPL (n = 256) and the control group (n = 70). Pathological characteristics and levels of gastrin 17 (G17), pepsinogen (PG) I and II and anti-*Helicobacter pylori* IgG were assessed in GPL cases, and the severity of intestinal metaplasia and gastric atrophy was scored by expert pathologists. **Results**: APCA positivity was observed in 18% of cases vs. 7% of controls (*p* = 0.033). Only 3/256 patients were previously diagnosed with AIG. The presence of APCA was associated with corpus-limited and extended GPL. A receiver operating curve analysis demonstrated that the G17 and PGI/II ratio could identify APCA-positive patients within GPL cases (AUC: 0.884). **Conclusions**: The prevalence of AIG is higher in patients with GPL but goes undiagnosed. Using G17 and PG I/II as diagnostic markers can help to identify patients with AIG and improve surveillance programs for patients with GPL.

## 1. Introduction

Gastric cancer (GC) is the sixth most common cancer worldwide, with an incidence that varies per geographic region. Although the global incidence of GC is declining, it is still the fourth cause of cancer-related mortality [1]. The strongest known risk factor for GC development is infection with the bacterium *Helicobacter pylori* (*H. pylori*) [2]. According to Correa’s cascade, GC development starts from *H. pylori*-induced inflammatory changes of the normal gastric mucosa, which progresses through chronic gastritis, chronic atrophic gastritis (AG) and gastric intestinal metaplasia (IM) to dysplasia and carcinoma cc [3]. Endoscopic surveillance of gastric precancerous lesions (GPL) such as AG or IM decreases the risk of developing invasive GC [4,5]. Thus, international guidelines on the management of GPL recommend that patients with extensive AG and/or IM should undergo endoscopic surveillance every 3 years [6,7,8,9].

Another cause of chronic gastritis is autoimmune gastritis (AIG), an immune-mediated disorder characterized with the destruction of gastric parietal cells secondary to the presence of anti-parietal-cell antibodies (APCA), leading to the loss of intrinsic factor (IF), which may result in development of pernicious anemia and reduced gastric acid production [10]. Patients with AIG, APCA or pernicious anemia have a three- to five-fold increased risk of developing GC [11,12,13], and a recent study suggests that AIG may replace *H. pylori* as the main driver of GC risk, in particular, in young women [10]. Indeed, the European Management of Precancerous conditions and lesions of the Stomach (MAPS) guideline was updated in 2019 to also include AIG, and now suggests that patients with AIG may benefit from endoscopic surveillance every 3–5 years, or 3 years in the presence of any GPL [9]. The American Gastroenterology Association Guidelines also suggest that interval endoscopic surveillance for AIG patients should be considered based on individualized assessment [14]. Nonetheless, the association between AIG and GC, as well as the optimal surveillance strategy for AIG remains controversial, one possible explanation for this is the underdiagnosis of AIG in clinical practice.

To date, there is no global consensus on the diagnostic criteria for AIG, and the method of diagnosis varies widely across different studies. One commonly used method (golden standard) is the assessment of the histopathologic manifestation including corpus-dominant lymphoplasmacytic inflammation, atrophy of the glandular layer and pseudopyloric/pancreatic or intestinal metaplasia [15]. However, in patients who undergo gastroscopy, biopsies are not always obtained, or sampling is not performed adhering to the recommended biopsy protocol, potentially affecting the accuracy of the diagnosis. In addition, antral gastritis due to concurrent *H. pylori* infection in AIG patients often confounds the correct diagnosis of AIG [16,17]. Thus, it is believed that AIG might be underdiagnosed in AG/IM patients due to similar pathological manifestations and the absence of typical symptoms [18]. The detection of APCA can help to confirm the presence of AIG. Furthermore, biomarkers for gastric function like gastrin 17b (G17), pepsinogen (PG) I, PGII and the I/II ratio [19] may also be helpful, as achlorhydria caused by the destruction of the parietal cells induces gastrin production by continuously stimulating gastric G-cells, while mucosal atrophy reduces PGI synthesis [20]. Vitamin B12 deficiency, caused by a lack of IF production, may be seen in the late stages of AIG. Despite the availability of several potential diagnostic tools, patients with GPL are not routinely screened for AIG. In addition, it is unclear to what extent identification of AIG using these markers is affected by the presence of *H. pylori*-mediated AG or IM.

Differentiating between GPL caused by AIG or any other etiology (i.e., *H. pylori*) is important as, at least according to European guidelines, identification of AIG will result in a recommendation for surveillance every 3 years even if GPL is seen at a single location, while these patients are excluded from further surveillance in the case of an *H. pylori*-associated gastritis. Nevertheless, the prevalence of AIG remains unclear in this patient population.

Therefore, we investigated the prevalence of AIG in a prospectively followed cohort of patients with GPL. In addition, we aimed to identify the clinical characteristics of AIG-associated IM and attempted to determine the diagnostic efficiency of gastric function biomarkers to optimize the detection of AIG in patients with GPL.

## 2. Materials and Methods

### 2.1. Study Population

The Progression and Regression of precancerous Gastric Lesions (PROREGAL) study is an ongoing prospective, multicenter study [21]. Initiated in 2009, it includes six hospitals (one academic and five regional) in the Netherlands and one regional hospital in Norway. All included patients are older than 18 years with a previous diagnosis of atrophic gastritis (AG), intestinal metaplasia (IM) and/or dysplasia of the gastric mucosa. Patients are excluded if they have a history of upper gastrointestinal surgery or gastric carcinoma or have a severe comorbidity limiting their expected survival to less than 2 years. We included 256 patients from the PROREGAL cohort in the current study, based on availability of serum samples. Demographic data and vitamin supplementation information were collected through questionnaires at baseline. Information about the concurrent with other autoimmune diseases was collected for APCA-positive patients. As *H. pylori* has been associated with AIG [22,23], and has even been suggested to induce AIG [24,25], we included a control cohort consisting of 70 consecutively included patients older than 18 years who were invited to undergo a urea breath test (UBT) for suspected *H. pylori* infection [26], thereby ensuring similar *H. pylori* prevalence in cases and controls. The study was approved by the Medical Ethical Review Committee of Erasmus MC (MEC-2009-090, MEC-2017-528), and informed consent was obtained from all included participants.

### 2.2. Serological Tests

Serum from patients enrolled in the PROREGAL study was collected at baseline and each endoscopy follow-up visit, while serum of control subjects was collected at the day of UBT. All samples were stored at −80 °C. Serum anti-parietal-cell antibodies (APCA) were pre-screened through a qualitative indirect immunofluorescent antibody test (IFT) employing commercial tissue sections (ImmuGlo™ Rat stomach slides, Immco Diagnostics, Buffalo, NY, USA) incubated with patient sera (diluted 1:10) followed by anti-human IgG-FITC (Inova Diagnostics Inc., San Diego, CA, USA). Samples testing positive with IFT were subsequently subjected to a H^+^K^+^ATPase-specific EliA^TM^ automated enzyme fluoroimmunoassay using the Phadia 250 system (Thermo Fischer Scientific, Freiburg, Germany) allowing quantitative statements for the concentration of APCA (U/mL). GastroPanel (Biohit Qyi, Helsinki, Finland) was used to identify the serum levels of PGI, PGII, G17 and anti-*H. pylori* antibodies, as per manufacturer instructions. Optical density (OD) measurements (450 nm) were performed with an infinite 200 pro ELISA reader (TECAN, Mannedorf, Switzerland). The normal range values for these parameters are APCA > 10 U/mL; PGI, 30–160 μg/L; PGII, 3–15 μg/L; PGI/PGII, 3–20; G17, 1–75 pmol/L; and anti-*H. pylori* antibodies < 30 EIU. Anti-*H. pylori* antibodies ≥ 30 EIU indicate that the patients had a previous *H. pylori* infection that was eradicated, or an active *H. pylori* infection without eradication.

### 2.3. Histopathology

All patients from the PROREGAL study underwent at least one surveillance endoscopy after the index endoscopy. During endoscopy, biopsy samples were collected according to the PROREGAL biopsy protocol from all the visible lesions and 5 standardized sites, including four-quadrant biopsies of the antrum, two from the lesser curvature and two from the greater curvature and two from cardia [27]. The retrieved tissues were fixed in formalin (10%) and embedded in paraffin. Histological assessment of biopsy specimens of each gastric region was retrieved from patient records, as assessed by the pathologists from the participating hospitals where the biopsies were collected, but who, at the time, were not specifically asked whether histological characteristics fitting with AIG were present. Given the purpose of this study, AIG-associated pathological features were re-evaluated in APCA-positive patients to confirm the diagnosis of AIG. The presence of AG and stage of IM were evaluated according to the modified OLGA and OLGIM staging systems.

### 2.4. Statistical Analysis

For the baseline demographics and characteristics, continuous variables were expressed as the mean ± standard deviation (SD) or median with the interquartile range (IQR). Categorical variables are presented as percentages. Differences in age, gender, APCA positivity and *H. pylori* infection history between the two cohorts were assessed using univariable binary logistic regression. The independent correlation of GPL with APCA positivity was verified by using a multivariable binary logistic regression analysis to adjust for age, gender and *H. pylori* infection. The distribution of GPL between patients with or without APCA was compared using the Chi-square test, while the differences of the OLGIM and OLGA score between these two groups were compared with the Mann–Whitney U test. Kendall’s tau-b was used to measure the correlation between the OLGIM score or OLGA score and APCA concentration. Differences in PGI, PGII and G17 and *H. pylori* IgG levels between 2 groups or between 4 groups were assessed with the Mann–Whitney U test or Kruskal–Wallis test (including Dunn–Bonferroni post hoc correction). An ROC curve was used to evaluate the diagnostic performance of PGI, PGII, the PGI/PGII ratio, G17 and *H. pylori* IgG. A *p* value < 0.05 was considered statistically significant. The statistical analysis was performed using SPSS statistical software (IBM SPSS Statistics 28.0.1.0 (142)).

## 3. Results

### 3.1. Prevalence of AIG Is Increased in Patients with Gastric Premalignant Lesions

In total, 326 subjects were included in our study, 256 GPL cases and 70 controls. The baseline characteristics of both groups are shown in Table 1. Patients were more often male (50% vs. 34%, *p* = 0.024), and were significantly older than controls (64 years (55–71) vs. 51 years (43–60), *p* < 0.001). The percentage of subjects with active or previous *H. pylori* infection was similar between the two groups (40% vs. 31%, *p* = 0.2). APCA positivity was seen for 51 individuals, 36 of whom were women. A significantly higher prevalence of APCA positivity was seen in the GPL group as compared to the control cohort (18% vs. 7%, *p* = 0.033). The multivariable logistic regression analysis showed that APCA positivity (adjusted OR = 3.76, 95% CI = −1.31 to 10.79; *p* = 0.013), age (adjusted OR = 1.07, 95% CI = −1.04 to 1.09; *p* < 0.001) and male gender (adjusted OR = 0.47, 95% CI = −0.26 to 0.86; *p* = 0.015) were independently associated with IM.

Only three of the APCA-positive GPL cases were previously identified with AIG. To confirm the presence of AIG features in APCA-positive patients among the GPL cases, the HE-stained slides were (re)assessed, showing Enterochromaffin-like (ECL) cell hyperplasia in 19 of 46 patients. Furthermore, more than half of patients presented with low vitamin B12 levels, while concomitant autoimmune disease was observed in 23.7% of cases (see Supplementary Table S1). Within the prospective PROREGAL study, patients were followed up with per study protocol irrespective of extent of AG/IM, and thus more frequently as compared to MAPSII guidelines. However, of the 46 APCA-positive patients, 19 presented with limited AG/IM lesions, which would have made them eligible for exclusion of further surveillance according to the MAPSII guidelines. Thus, without direct confirmation of an AIG diagnosis, 44.2% (19/43) of patients who should have been kept under surveillance because of the presence of AIG would have been lost to follow up.

### 3.2. AIG in GPL Patients Is Associated with Gastric Location, but Not Severity of GPL

To further identify the characteristics of APCA-positive GPL patients, we compared the distribution of precancerous lesions within the gastric mucosa at the time of serum collection, between individuals with or without APCA. We found that 51.2% of APCA-positive patients have extended GPL, which is significantly more than in APCA-negative patients. In patients with restricted GPL, corpus involvement was more frequently identified in APCA-positive patients (39.0% vs. 11.2%), while in APCA-negative subjects, GPL was more frequently located in the antrum (9.8% vs. 59.7%, *p* < 0.001, Table 2).

**Figure 1:**
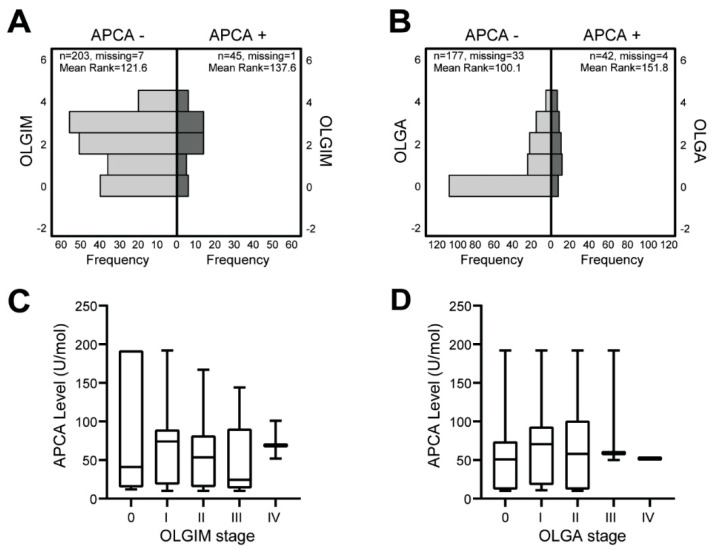
**Association between anti-parietal cell antibodies and severity of intestinal metaplasia and gastric atrophy**. (**A**) No differences in Operative Link for Gastric Intestinal Metaplasia Assessment (OLGIM) stage were seen for cases with GPL positivity for anti-parietal cell antibodies (APCA) and those negative for APCA (*p* = 0.16). (**B**) Operative Link of Gastritis Assessment (OLGA) score of zero was more often seen for GPL cases without APCA (*p* < 0.001). (**C**) Serum concentration of APCA did not correlate with OLGIM score (r = −0.06; *p* = 0.62). (**D**) Serum concentration of APCA did not correlate with OLGA score (r = 0.14; *p* = 0.31).

The OLGIM score was not affected by either APCA status (*p* = 0.16) or concentration of APCA (r = −0.06; *p* = 0.62; Figure 1A,C). However, the OLGA stage was lower in patients without APCA (*p* < 0.001), although APCA levels did not correlate to severity of atrophy (r = 0.14; *p* = 0.31; Figure 1B,D). Sampling error may occur during endoscopy and as such, the absence of IM during follow up is not always indicative of regression or absence of disease. Therefore, APCA was also compared to the worst OLGIM and OLGA score seen at any time during follow up, with similar results (Supplementary Figure S1).

### 3.3. AIG in the Context of Precancerous Gastric Lesions Is Associated with Serum Markers Indicative of Parietal Cell Loss

As destruction of parietal cells results in a change of serological biomarkers, we investigated whether gastrin and pepsinogen levels would identify AIG patients in the context of GPL. In APCA-positive GPL cases, the median levels of PGI, PGII and their ratio were 14.2 µg/L, 16.7 µg/L and 0.8, respectively, of which PGI and the PGI/II ratio were significantly lower as compared to APCA-negative subjects (Table 3). Conversely, in APCA-positive GPL cases, the median G17 levels were significantly higher than in APCA-negative cases (107.5 vs. 4.9 pmol/L; *p* < 0.001). Anti-*H pylori* IgG levels were similar between the two groups.

**Figure 2:**
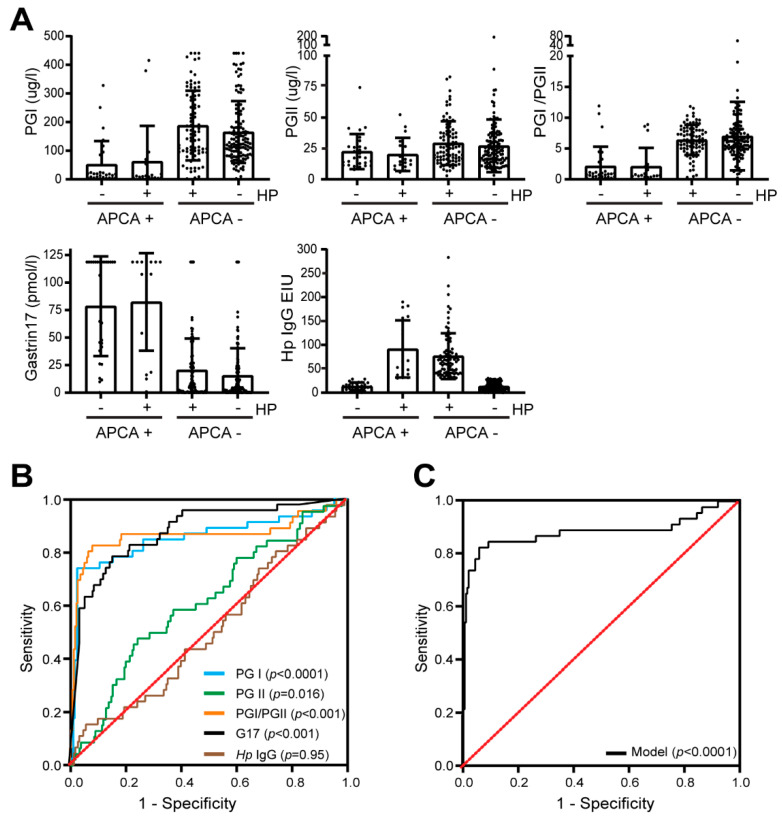
**Biomarker analysis for detection of autoimmune gastritis**. (**A**) Serum level of PGI, PGII, PGI/II ratio, G17 and *H. pylori* IgG in patients with only autoimmune gastritis (AIG), patients with AIG and previous or active *H. pylori* infection, patients with only *H. pylori* infection and patients with no *H. pylori* infection or AIG. (**B**,**C**) Receiver operating characteristic (ROC) curve analysis of PGI, PGII, PGI/PGII ratio, G17 and *H. pylori* IgG for discrimination of patients with and without AIG.

We next divided the GPL patients into four groups, based on their history of *H. pylori* infection and APCA status. This analysis showed that G17, PGI and PGII levels are dependent on APCA positivity rather than the history of *H. pylori* infection, suggesting that using these biomarkers to screen for AIG is a viable option and that history of *H. pylori* infection will not affect their diagnostic efficacy (Figure 2A).

We then investigated the diagnostic accuracy of GastroPanel in predicting AIG with an ROC analysis. While the diagnostic ability of PGII and *H. pylori* IgG was low (area under the ROC curves (AUC) of 0.613 [95% CI = 0.551–0.673] and 0.503 [95% CI = 0.440–0.566], respectively), individual measurements like PGI, the PG I/II ratio and G17 showed good diagnostic accuracy, with an AUC of 0.856 (95% CI = 0.807–0.896) for PGI, 0.865 (95% CI = 0.817–0.904) for the I/II ratio and 0.873 (95% CI = 0.825–0.911) for G17 (Figure 2B). By combining the PGI/II ratio and G17, the AUC increased to 0.884 (95% CI = 0.838–0.920), based on Youden’s J statistic, with an optimal cut-off of >0.27, and a corresponding sensitivity of 80.4% (95% CI = 66.1–90.6) and a specificity of 94.2% (95% CI = 90.2–97.0), indicating that this panel could be a valuable supplementary tool in the detection of AIG among patients with GPL (Figure 2C).

## 4. Discussion

In this study, we showed that the prevalence of AIG is 18% in a population with GPL, compared to a 7% prevalence in controls. While previous studies reported that IM is relatively common in AIG cohorts [28,29,30], we demonstrate the reverse, i.e., IM is associated with an increased prevalence of AIG. This is of relevance, as when IM or AG is found, it is conceivable that clinicians may not think to test further for the etiology of these mucosal abnormalities, in particular, if a history of *H. pylori* is known. Nevertheless, the accurate diagnosis of AIG within a population with GPL is essential to determine their endoscopic follow-up strategy, particularly since European guidelines suggest that endoscopic follow up is required every 3 years for patients with AIG and AG/IM limited to the gastric antrum or corpus (conditions that, without AIG, would qualify patients for release from surveillance) [9]. In our study, only three patients were diagnosed with AIG prior to the start of the study, which means that AIG may go underdiagnosed in patients with GPL. Possible explanations are that AIG is asymptomatic in its early stages, that histological images may resemble AG in the context of IM and that most patients are diagnosed only when pernicious anemia is present. Furthermore, 44.2% of AIG patients in our cohort would have been dismissed from surveillance based on limited severity of endoscopy findings when following the MAPS-II guideline, if APCA status had not been known. It is of interest to note that longitudinal assessment of AIG patients without IM at presentation showed development of IM in some of these patients within a 10-year follow up [31]. Furthermore, not all individuals already have mucosal structural abnormalities at the time of APCA detection [32]. Such mucosal alterations may develop over time, something that may occur in up to 50% of ‘potential’ APCA-positive patients [33]. It is possible that IM is missed during a routine endoscopy at presentation, or that AIG may lead to the IM development, which has not been studied thus far. In any case, an accurate and timely diagnosis of AIG is imperative to provide patients with optimal care.

Nowadays, the most reliable diagnosis of AIG rests on endoscopic or pathological findings and serum markers of autoantibodies targeting gastric parietal cell H^+^/K^+^ ATPase (APCA) or intrinsic factor (AIFA). The classical pathological feature of AIG is inflammation predominantly presented at oxyntic mucosa, but such an unequivocal presentation is rare, especially when concurrent *H. pylori* infection induces antral gastritis [15]. We found that 39% of AIG patients have corpus-limited atrophy or IM, but that 51.2% of AIG patients have extended gastric atrophy or IM, hampering identification of AIG based on pathology reports alone. In addition, the pathological features may not always reflect the APCA status. A prospective follow up of 25 APCA-positive patients showed development of gastric mucosa atrophy after 5 years in only 6 patients, whereas Nishizawa et al. found that the end stage of AIG may be accompanied with a loss of APCA serum levels. Gender might be able to help us identify AIG, as most APCA-positive patients (67.4%) in our study were female, consistent with other studies showing a female predominance of AIG [34,35]. Additionally, some studies have classified patients as having AIG based on the presence of low serum vitamin B12. However, vitamin B12 supplementation is common (at least in our cohort), which may complicate its use for diagnostic purposes. Several studies have shown that measuring serum levels of PGI, PGII, G17 and anti-*H. pylori* antibodies can be used to diagnose atrophic gastritis and intestinal metaplasia [36,37,38,39]. A study from Turkey showed that the PG1/PG2 ratio may be useful to identify both autoimmune and environmental atrophic gastritis [40]. A Japanese group showed that high G17 and low PG I/II ratios could be used to identify AIG in a cross-sectional cohort of patients undergoing upper endoscopy [41]. Perhaps the most closely related to our study is a study from France, in which the authors show that the PG I/II ratio can distinguish AIG-mediated atrophy from *H. pylori*-induced atrophic gastritis [42]. However, this latter study did not take G17 levels into account, and none of these studies reported on the usefulness of GastroPanel for the diagnosis of AIG within a cohort of patients with GPL. Our results show that regardless of the history of *H. pylori* infection, the levels of PG1, PG2 and G17 still significantly differentiated cases with APCA from those without. Thus, PG1, PGII and G17 measurement might be a reliable tool to help to identify AIG in patients with precancerous gastric lesions.

This study has real strengths. First, by prospectively following our cohort of patients with premalignant lesions, we were able to accurately link the presence of APCA to the course of disease. Secondly, this cohort is one of the largest prospective cohorts of patients with GPL to date and currently has a follow-up time of over 10 years.

We also acknowledge several limitations to our study. First, we were unable to endoscopically confirm the absence of GPL in our control cohort. Thus, it remains unclear whether APCA positivity in this group reflects lack of specificity of the antibody test or whether the APCA-positive individuals in this population indeed suffer from AIG. However, we performed both indirect immunofluorescence as well as EliA to define APCA positivity, limiting the chance of false positivity. Second, the control cohort used consisted of individuals undergoing UBT. Thus, this population is likely enhanced for patients with dyspeptic symptoms and may therefore harbor a higher percentage of AIG patients as compared to the prevalence in the general population (~0.5–4.5%) [43]. However, this would suggest an underestimation of the difference in the prevalence of AIG between otherwise healthy individuals and those identified with premalignant lesions, and would therefore not substantially alter the conclusions of this study.

Overall, our data show that the prevalence of AIG is increased in patients presenting with GPL and may be subject to underdiagnoses. Awareness for AIG testing should be raised among both pathologists and gastroenterologists in order to optimize surveillance strategies.

## Figures and Tables

**Table 1:** Baseline characteristics of study population and association between age, gender, APCA positivity, *H. pylori* infection history and GPL.

Parameter	GPL Cases (n = 256)	Controls (n = 70)	Crude OR (95% CI) ^a^	*p* Value	Adjusted OR (95% CI) ^b^	*p* Value
Age at index endoscopy, median (IQR), years	64 (55–71)	51 (43–60)	1.07 (1.04–1.09)	<0.001	1.07 (1.04–1.09)	<0.001
Gender, male (%)	127 (50)	24 (34)	0.53 (0.31–0.92)	0.024	0.47 (0.26–0.86)	0.015
Positivity of APCA, + (%)	46 (18)	5 (7)	2.84 (1.09–7.47)	0.033	3.76 (1.31–10.79)	0.013
*H. pylori* infection, yes (%)	102 (40)	22 (31)	1.45 (0.82–2.54)	0.2		

^a^ Crude OR (95% CI) and *p* value were determined with binary logistic regression. ^b^ Adjusted OR (95% CI) and *p* value were determined with multivariate logistic analysis, adjusted for age and gender. Abbreviations: OR, odds ratio; CI, confidence interval; IQR, interquartile range; APCA, anti-parietal-cell antibodies; GPL, gastric precancerous lesion (includes gastric atrophy and gastric intestinal metaplasia).

**Table 2:** Distribution of precancerous lesion in the gastric mucosa of patients with or without anti-parietal-cell antibodies.

Index Endoscopy	GPL Cases				*p* Value
	APCA Positive (n = 43)		APCA Negative (n = 174)		
*N*	n	%	n	%	
GPL in Antrum	4	9.8	104	59.7	<0.001
GPL in Corpus	15	39.0	19	11.2	
Extended GPL	24	51.2	51	31.1	

**Table 3:** Results of GastroPanel testing in patients with or without anti-parietal-cell antibodies.

Parameter	GPL Cases		*p* Value
	APCA Positive	APCA Negative	
*N*	46	210	
GastroPanel			
PGI, µg/L	14.2 (5.4–57.7)	139.0 (92.5–249.5)	<0.001
PGII, µg/L	16.7 (11.4–26.8)	22.7 (15.3–36.2)	0.16
PGI/PGII	0.8 (0.4–2.1)	6.2 (4.9–8.1)	<0.001
G17, pmol/L	107.5 (40.6–118.6)	4.9 (0.8–23.3)	<0.001
*H. pylori* IgG, EIU	21.1 (10.8–49.1)	23.0 (9.7–49.8)	0.95

Values are shown as median (interquartile range). Abbreviations: PGI, pepsinogen I; PGII, pepsinogen II; G17, gastrin 17b.

## Data Availability

Data is contained within the article or supplementary material.

## Supplementary Materials

The following supporting information can be downloaded at: https://www.mdpi.com/article/10.3390/jcm12196152/s1, Table S1. Detailed clinical findings of APCA positive cases. Figure S1. Serum concentration of APCA did not correlate with the worst OLGIM score detected during longitudinal follow-up (A, r = −0.1; *p* = 0.39) or the worst OLGA stage (B, r = 0.01; *p* = 0.96).
