# Supplementary material for: Increased Prevalence of Autoimmune Gastritis in Patients with a Gastric Precancerous Lesion
Source: J Clin Med. 2023 Sep 23;12(19):6152. doi: 10.3390/jcm12196152 (PMC10573100; doi:10.3390/jcm12196152)
Supplement: Supplementary file 1 [file jcm-12-06152-s001.zip › jcm-2568414-SI.pdf]

Supplementary

Table S1. Detailed clinical findings of APCA positive cases.

Characteristics	Autoimmune gastritis (N = 46)
**Diagnosis of H. pylori infection**	
⩾ 30 EIU / < 30 EIU	18/28 (39.1%/60.9%)
**Associated disorders**	
Hashimoto's thyroiditis, N (%)	7 (15.2%)
Graves' disease, N (%)	2 (4.3%)
Rheumatoid arthritis, N (%)	2 (4.3%)
Low Vitamin B12, N (%) *	25 (54.3%)
Pernicious anemia, N (%) ɫ	7 (15.2%)
**Associated pathology feature**	
Enterochromaffin-like cell hyperplasia, N (%)	19 (41.3)

*Low vitamin B12 were defined as vitamin B12 < 400 pmol/L
ɫVitamin B12 deficiency were defined as vitamin B12 < 145 pmol/L

Supplementary Figure S1

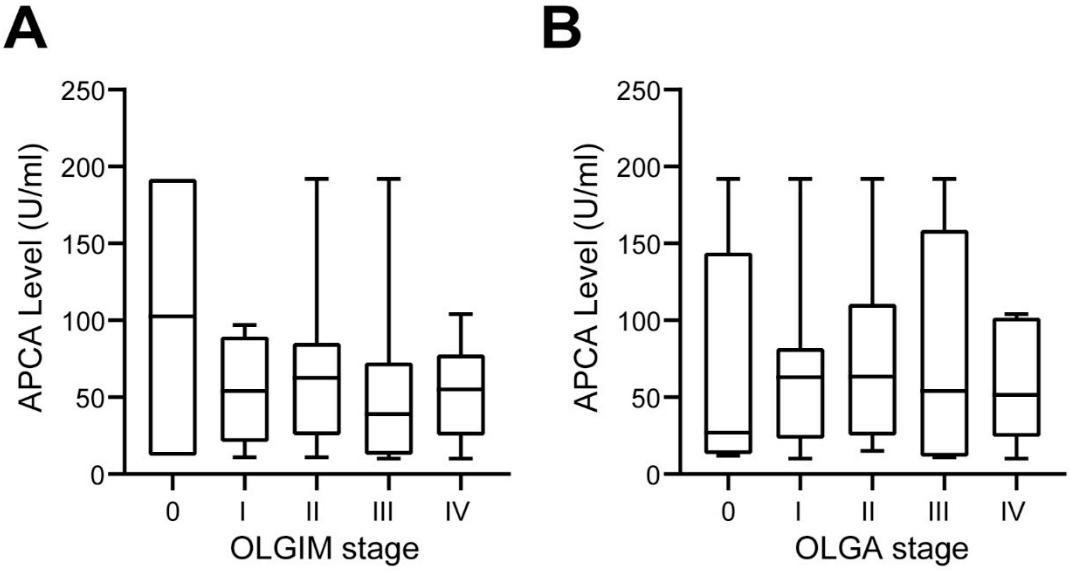

Supplementary Figure S1. Serum concentration of APCA did not correlate with the worst OLGIM score detected during longitudinal follow-up (A, r=-0.1; p=0.39) or the worst OLGA stage (B, r=0.01; p=0.96).
